# Supplementary material for: Cryo-EM Structures of the SARS-CoV-2 Endoribonuclease Nsp15
Source: bioRxiv. 2020 Aug 11:2020.08.11.244863. Preprint. [Version 1] doi: 10.1101/2020.08.11.244863 (PMC7427136; doi:10.1101/2020.08.11.244863)
Supplement: Supplement 1 [file media-1.pdf]

Supplementary Materials for

Cryo-EM Structures of the SARS-CoV-2 Endoribonuclease Nsp15

Authors

Monica C. Pillon[1,*,†], Meredith N. Frazier[1,†], Lucas B. Dillard[2,†], Jason G. Williams[3], Seda Kocaman[1], Juno M. Krahn[2], Lalith Perera[2], Cassandra K. Hayne[1], Jacob Gordon[1], Zachary D. Stewart[1], Mack Sobhany[1], Leesa J. Deterding[3], Allen L. Hsu[2], Venkata P. Dandey[2], Mario J. Borgnia[2], and Robin E. Stanley[1,*]

Affiliations

[1]Signal Transduction Laboratory, National Institute of Environmental Health Sciences, National Institutes of Health, Department of Health and Human Services, 111 T. W. Alexander Drive, Research Triangle Park, NC 27709, USA

[2]Genome Integrity and Structural Biology Laboratory, National Institute of Environmental Health Sciences, National Institutes of Health, Department of Health and Human Services, 111 T. W. Alexander Drive, Research Triangle Park, NC 27709, USA

[3]Epigenetics and Stem Cell Biology Laboratory, National Institute of Environmental Health Sciences, National Institutes of Health, Department of Health and Human Services, 111 T. W. Alexander Drive, Research Triangle Park, NC 27709, USA

[†]M.C.P., M.N.F., and L.B.D. contributed equally to this work and are co-first authors

*Co-corresponding authors: monica.pillon@nih.gov; robin.stanley@nih.gov

This PDF file includes:

Figures S1 to S9
Tables S1 to S2
Legends for Movies S1 to S4

Other Supplementary Material for this manuscript includes the following:

Movies S1 to S4

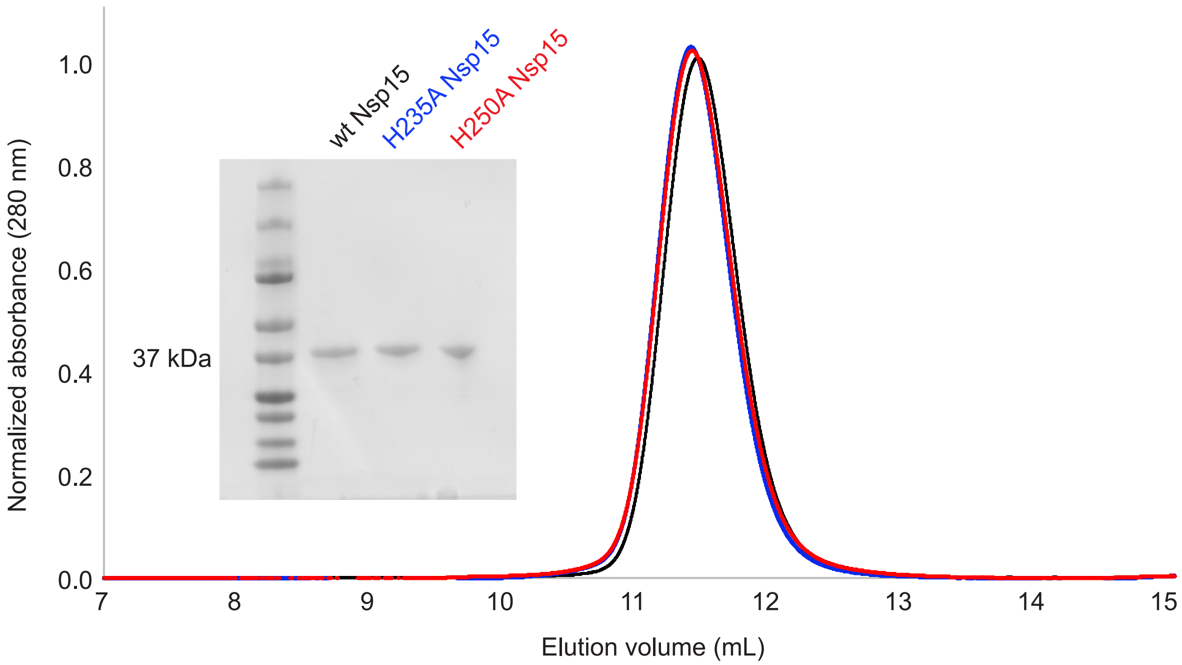

Fig. S1. Purification of recombinant SARS-CoV-2 Nsp15 variants. Purified Nsp15 variants wild-type (black), H235A (blue), and H250A (red) were resolved over a Superdex-200 increase 10/300 GL gel filtration column using SEC buffer. Inset is an SDS-PAGE analysis of recombinant Nsp15 wild-type (wt), H235A, and H250A variants.

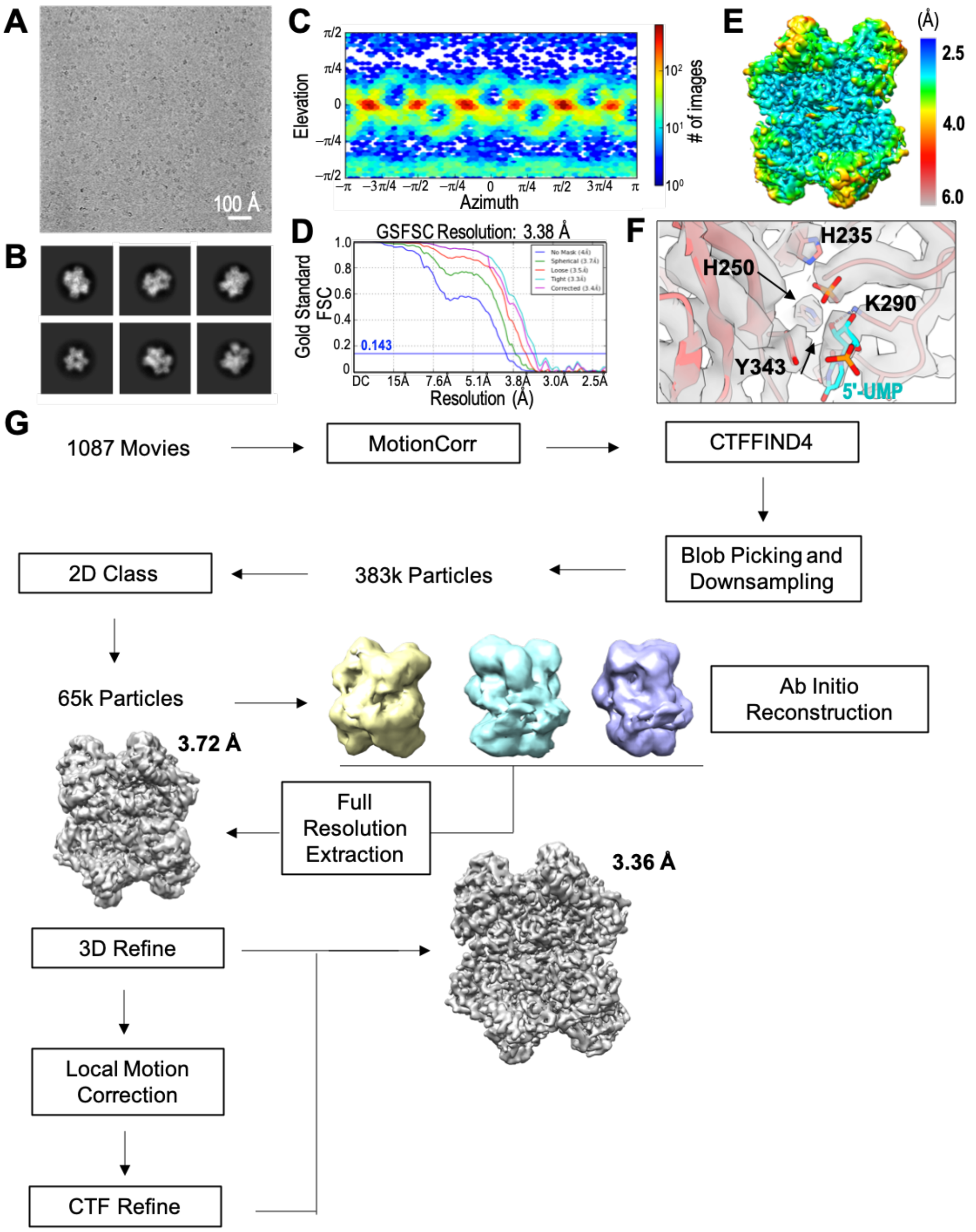

Fig. S2. Overview of cryo-EM processing scheme for UTP-bound Nsp15. (A) A representative micrograph of wt-Nsp15 in the presence of excess UTP in vitreous ice and **(B)** selected 2D classes generated from 1087 movies collected from an UltrAuFoil R1.2/1.3 300 mesh grid. **(C)** Angular distribution of UTP-bound wt-Nsp15 particles. **(D)** Fourier shell correlation (FSC) curve for the UTP-bound Nsp15 reconstruction. The overall resolution is 3.38 Å according to the FSC 0.143 criteria (*1, 2*). **(E)** Cryo-EM reconstruction of UTP-bound Nsp15 colored based on local resolution calculated using cryoSparc v2 (*3*). **(F)** Cryo-EM density for Nsp15 endoU active site (gray) with the corresponding model shown as a cartoon with individual active site residues, 5′-UMP, and modeled phosphate shown as sticks. **(G)** Cryo-EM processing workflow. Picked particles (383,271) were subjected to 2D classification, 3D classification, and refinement prior to local refinement of a single Nsp15 protomer in cryoSPARC v2 (*3*).

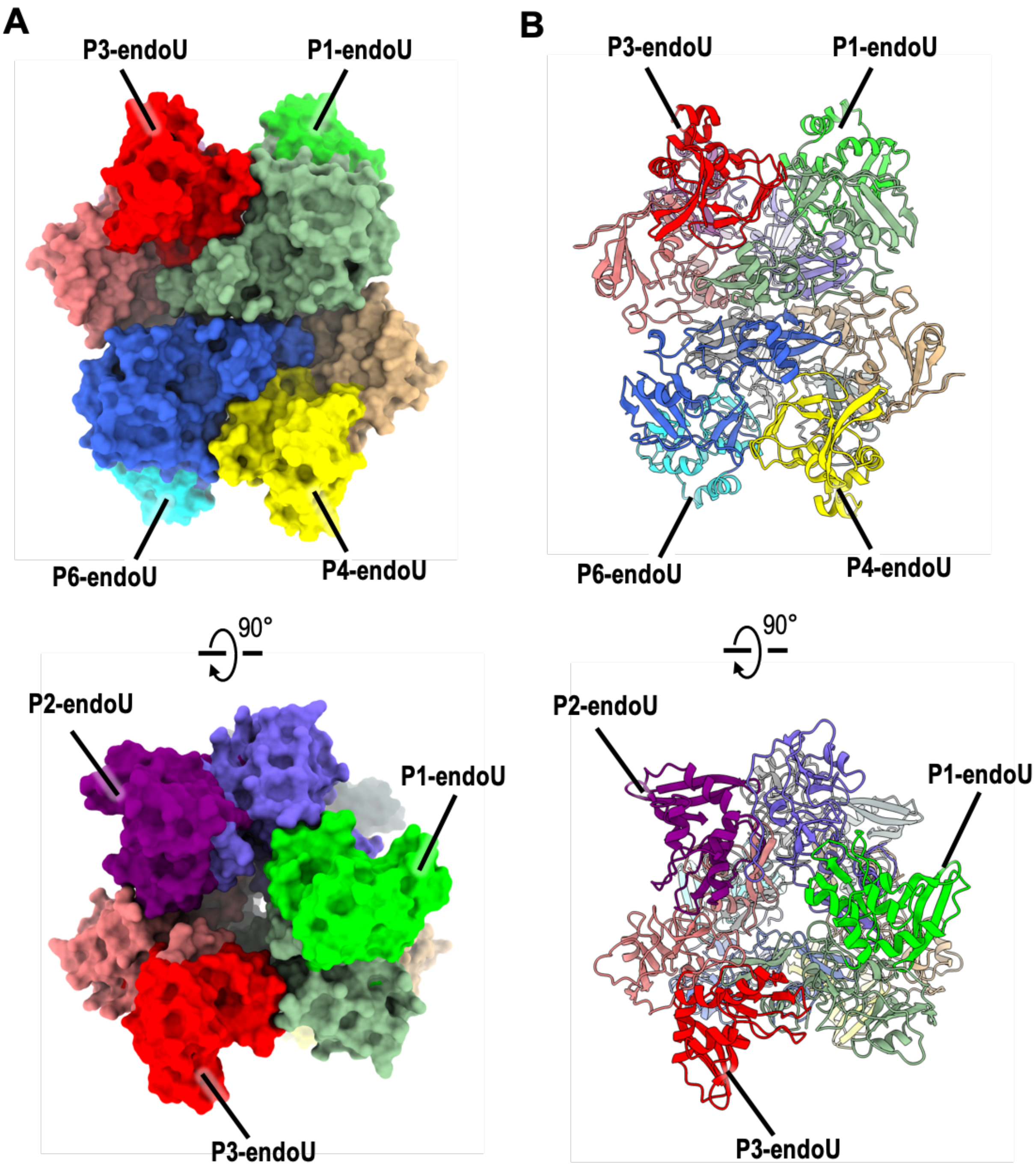

Fig. S3. Arrangement of Nsp15 endoU domains. Orthogonal views of **(A)** surface rendering and **(B)** cartoon model of hexameric Nsp15. The Nsp15 ND and MD domains are colored as seen in Fig. 2B. The endoU domains of six Nsp15 protomers (P1 to P6) are colored in bright hues of green, purple, red, yellow, gray, and cyan, respectfully.

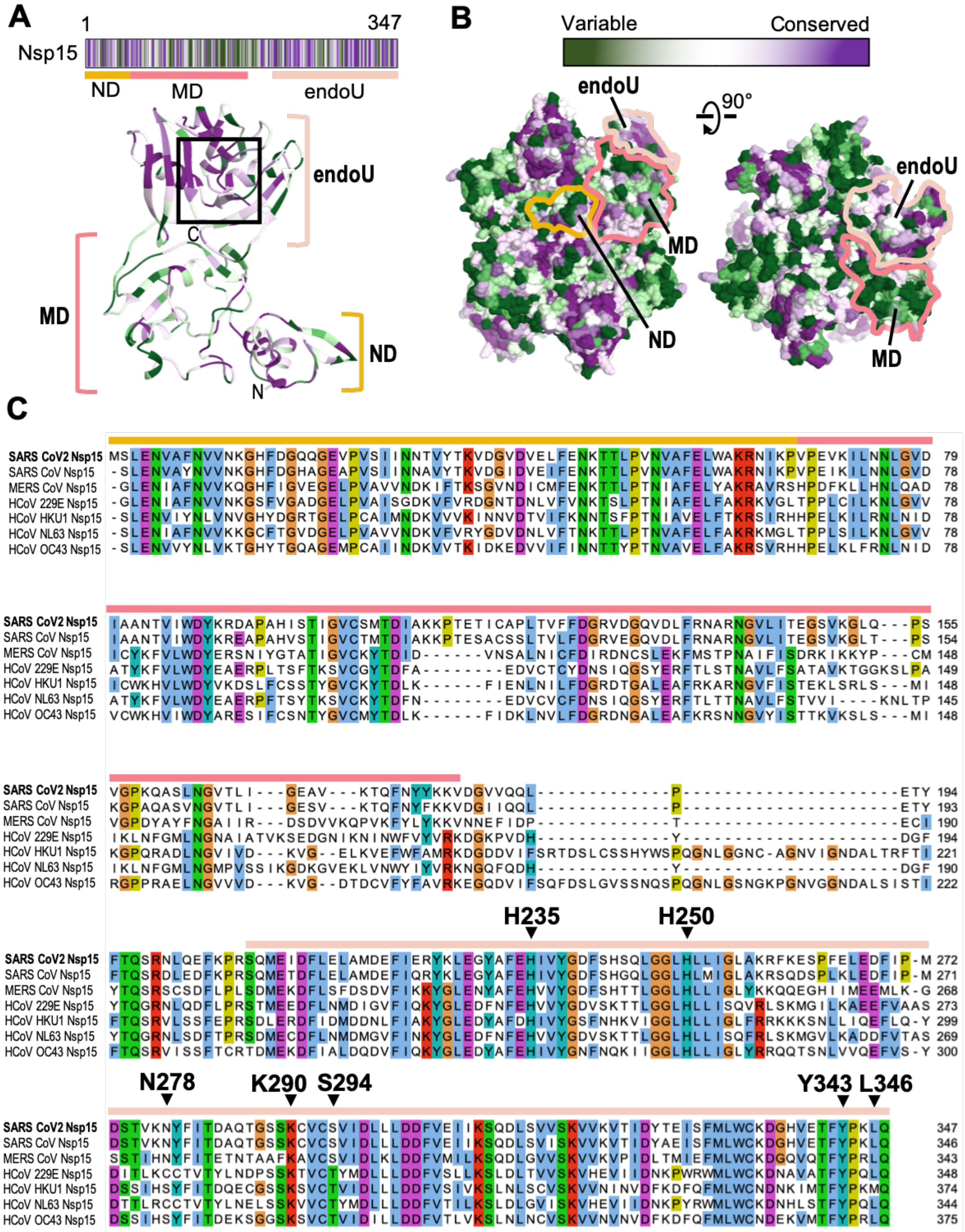

Fig. S4. Sequence alignment and conservation of Nsp15. (A) Consurf analysis (http:// consurf-hssp.tau.ac.il) of Nsp15 homologues plotted with respect to its domain architecture. Conserved and variable amino acid residues are colored as purple and green bars, respectively. The SARS-CoV-2 Nsp15 model of the protomer is shown as a cartoon and colored based on amino acid residue conservation. **(B)** Surface representation of hexameric Nsp15 illustrating conserved (purple) to variable (green) residues based on the Consurf analysis. The ND, MD, and endoU domains are outlined in orange, red, and beige, respectfully. **(C)** Sequence alignment of Nsp15 performed by PROMAL3D (*4*) and illustrated by JalView (*5*). Black arrowheads mark the position of Nsp15 endoU residues located within the active site.

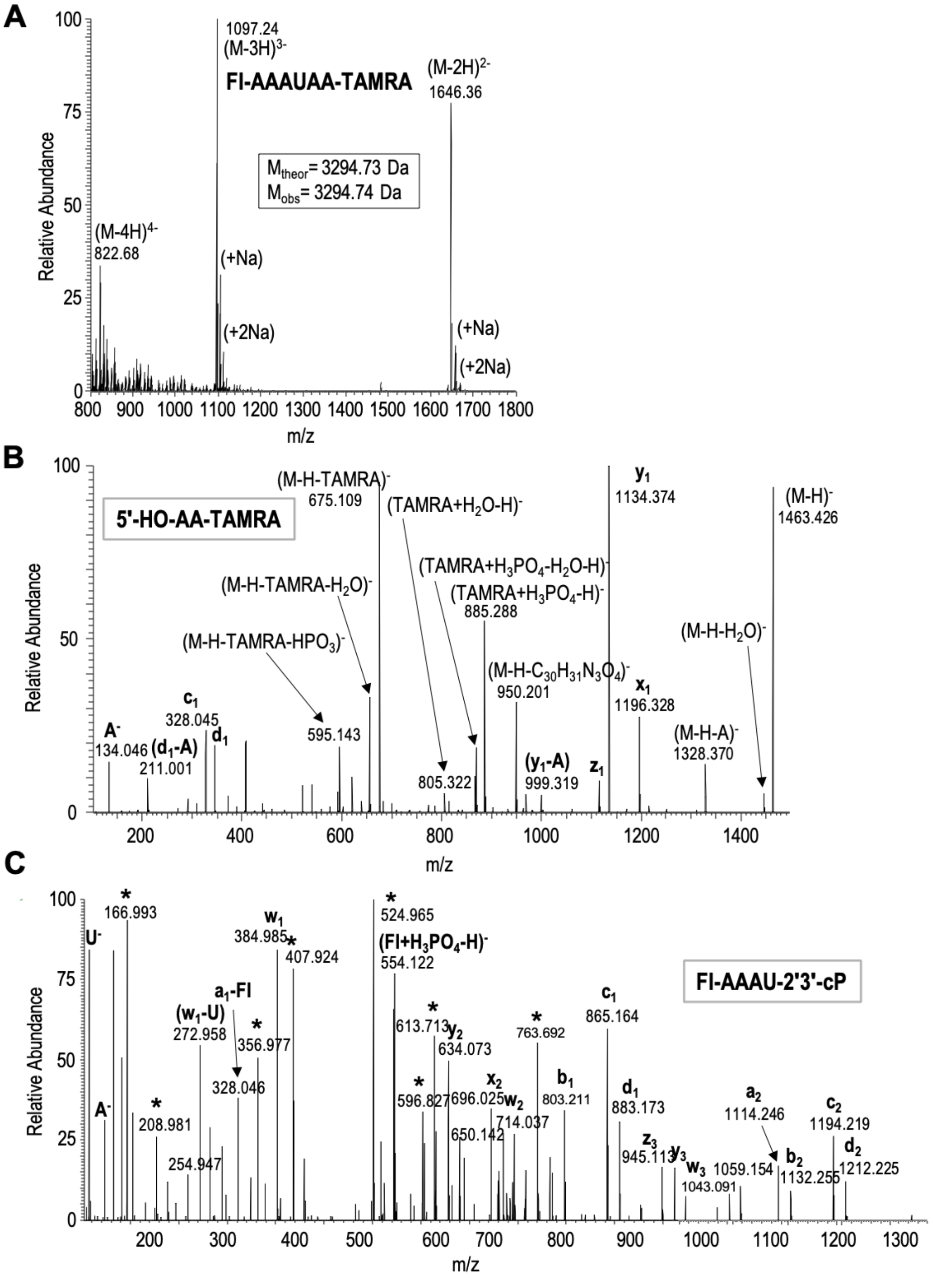

Fig. S5. RNA analysis using mass spectrometry. (A) The MS spectrum of the uncut FI-AAAUAA-TAMRA synthetic oligonucleotide shows doubly and triply charged species that correspond in mass to the theoretical mass of the intact oligonucleotide. **(B)** The MSMS spectrum of the m/z 1463.42 ion yields extensive fragmentation (*6*) that confirms the identity of 5′-HO-AA-TAMRA cleavage product. **(C)** The MSMS spectrum of the m/z 914.14 ion yields extensive fragmentation (*6*) that confirms the identity of FI-AAAU-2′3′-cP cleavage product. This spectrum is chimeric due to a coeluting, co-isolating molecule(s). Peaks labeled with asterisks (*) arise from coeluting, co-isolating species as determined by extracted ion chromatograms generated for the various fragment ions in the 914.14 channel. Fragment ions that have a distinct peak at 6.2 minutes were assumed to arise from the 5′-FI-AAAU-2′3′-cP cleavage product.

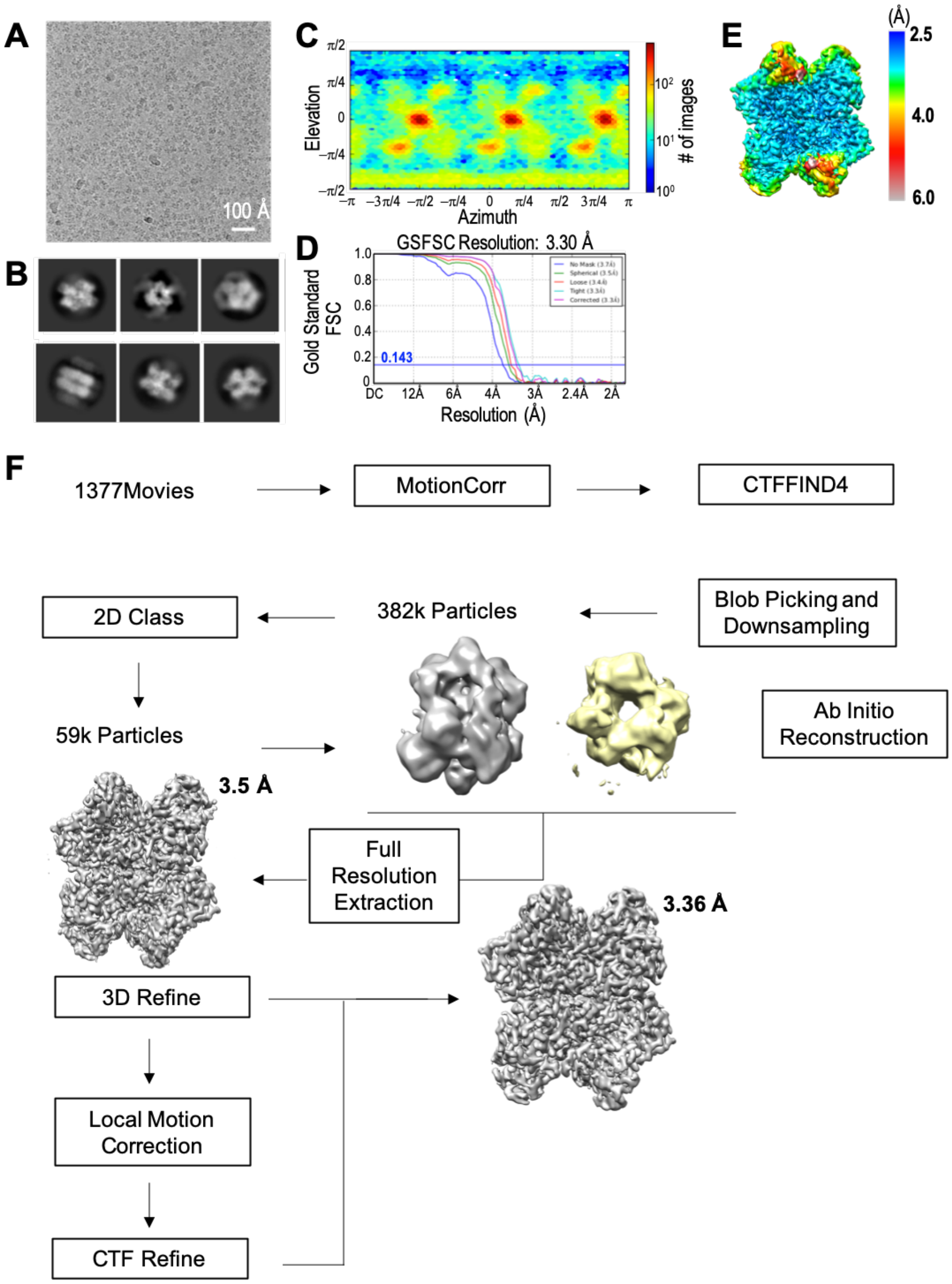

Fig. S6. Overview of cryo-EM processing scheme for apo-state Nsp15 H235A dataset i. (A) A representative micrograph of apo-state Nsp15 H235A in vitreous ice and **(B)** selected 2D classes generated from 1377 movies collected from an UltrAuFoil R1.2/1.3 300 mesh grid. **(C)** Angular distribution of apo-state Nsp15 H235A particles. **(D)** Fourier shell correlation (FSC) curve for the apo-state Nsp15 H235A reconstruction. The overall resolution is 3.30 Å according to the FSC 0.143 criteria (*1, 2*). **(E)** Cryo-EM reconstruction of apo-state Nsp15 H235A colored based on local resolution calculated using cryoSparc v2 (*3*). **(F)** Cryo-EM processing workflow. Picked particles (382,348) were subjected to 2D classification, 3D classification, and refinement prior to local refinement of a single Nsp15 protomer in cryoSPARC v2 (*3*).

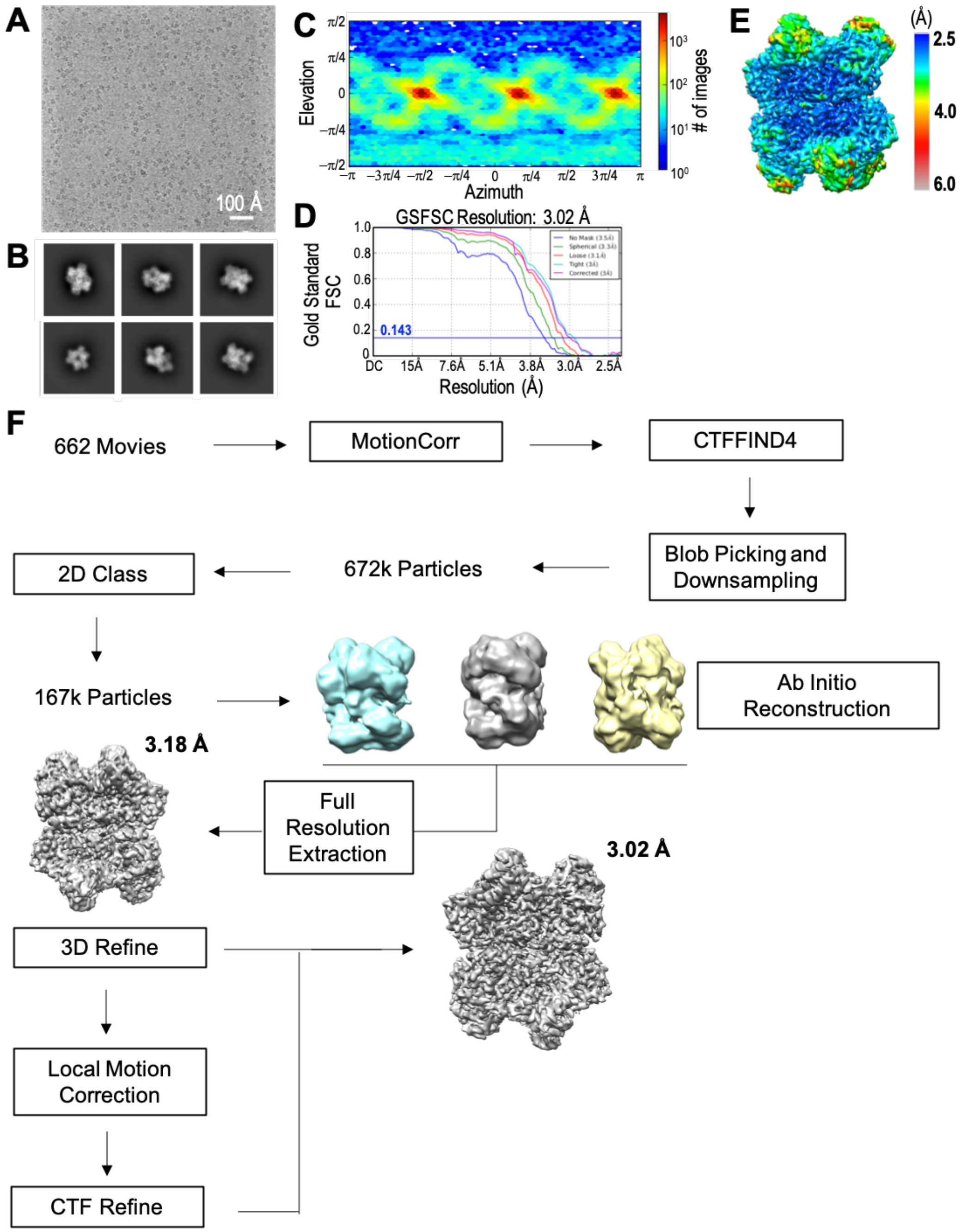

Fig. S7. Overview of cryo-EM processing scheme for apo-state wt Nsp15. (A) A representative micrograph of apo-state wt-Nsp15 in vitreous ice and **(B)** selected 2D classes generated from 662 movies collected from an UltrAuFoil R1.2/1.3 300 mesh grid. **(C)** Angular distribution of apo-state wt-Nsp15 particles. **(D)** Fourier shell correlation (FSC) curve for the apo-state Nsp15 reconstruction. The overall resolution is 3.02 Å according to the FSC 0.143 criteria (*1, 2*). **(E)** Cryo-EM reconstruction of apo-state Nsp15 colored based on local resolution calculated using cryoSparc v2 (*3*). **(F)** Cryo-EM processing workflow. Picked particles (672,879) were subjected to 2D classification, 3D classification, and refinement prior to local refinement of a single Nsp15 protomer in cryoSPARC v2 (*3*).

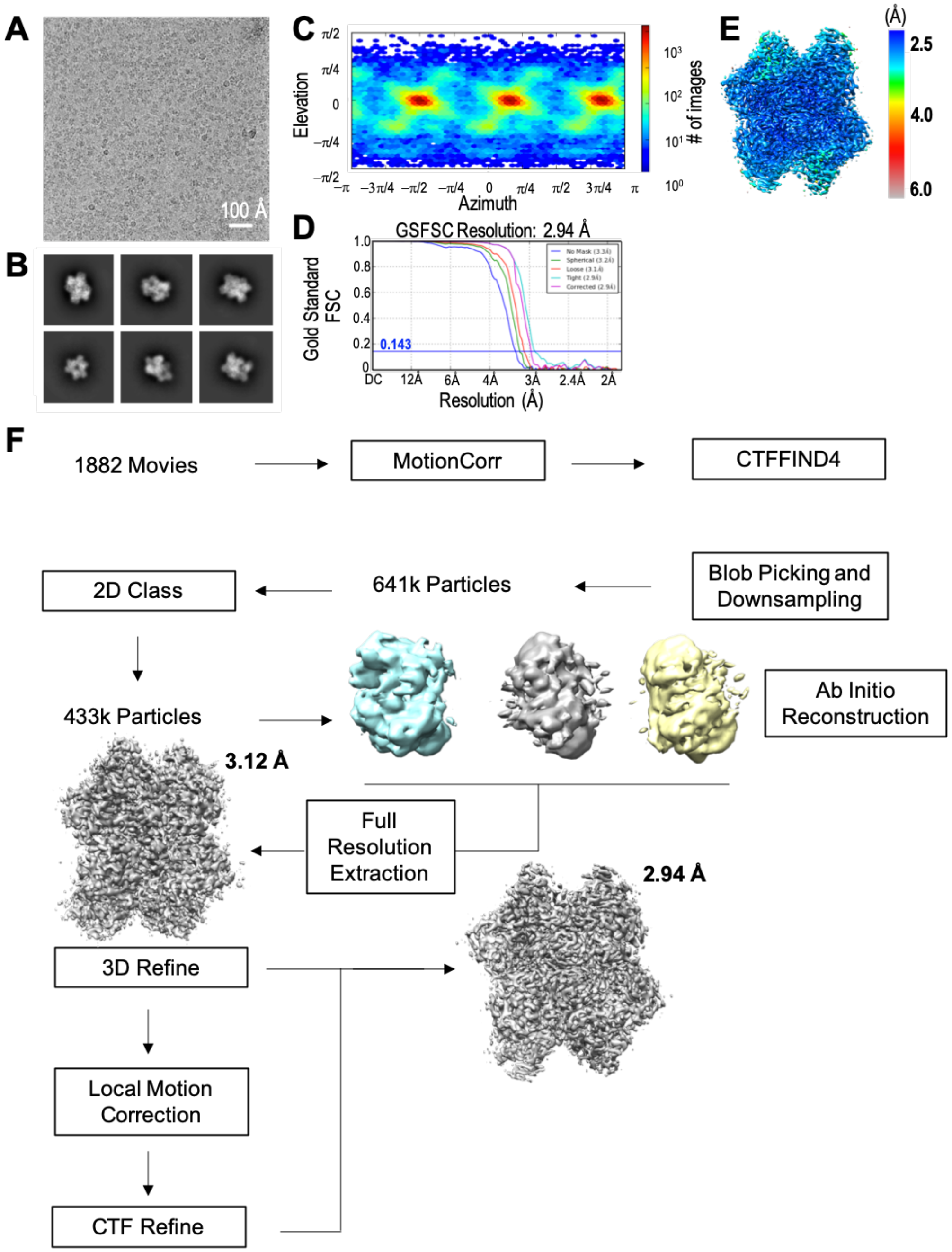

Fig. S8. Overview of cryo-EM processing scheme for apo-state Nsp15 H235A dataset ii. (A) A representative micrograph of apo-state Nsp15 H235A in vitreous ice and **(B)** selected 2D classes generated from 1882 movies collected from an UltrAuFoil R1.2/1.3 300 mesh grid. **(C)** Angular distribution of apo-state Nsp15 H235A particles. **(D)** Fourier shell correlation (FSC) curve for the apo-state Nsp15 reconstruction. The overall resolution is 2.94 Å according to the FSC 0.143 criteria (*1, 2*). **(E)** Cryo-EM reconstruction of apo-state Nsp15 H235A colored based on local resolution calculated using cryoSparc (*3*). **(F)** Cryo-EM processing workflow. Picked particles (641,748) were subjected to 2D classification, 3D classification, and refinement prior to local refinement of a single Nsp15 protomer in cryoSPARC v2 (*3*).

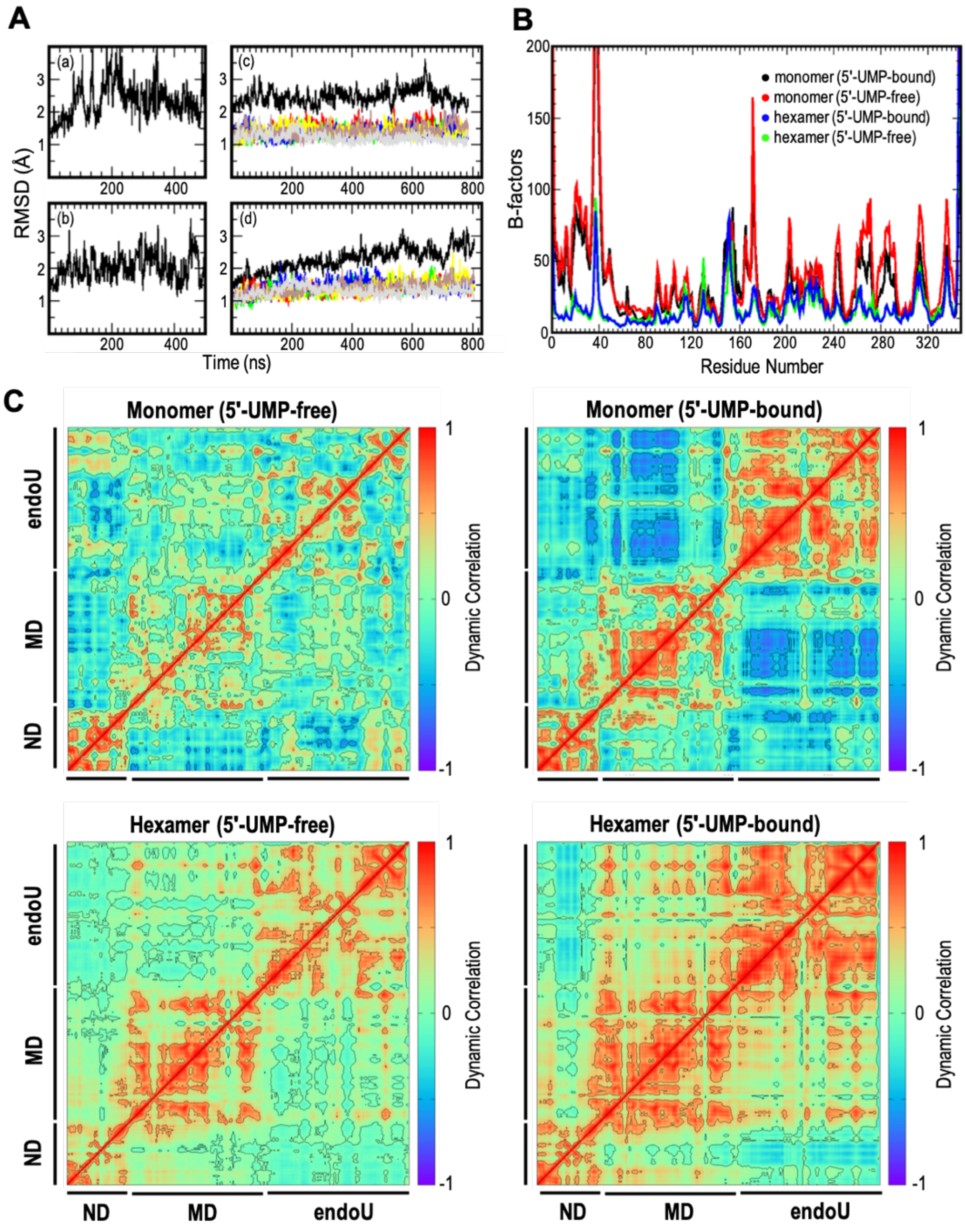

Fig. S9. Molecular dynamics of Nsp15. (A) Root mean square deviations (RMSDs) of (a) a substrate-free monomer, (b) 5′-UMP-bound monomer, (c) a substrate-free monomer from the hexamer (black= hexamer RMSD; colored plots= individual monomer RMSDs), and (d) a 5′-UMP-bound monomer from the hexamer (black= hexamer RMSD; colored plots= individual monomer RMSDs). Individual monomer RMSDs from hexamers systems are smaller when compared with the values of monomer systems, due to subdued dynamics of monomer in the hexameric configurations. **(B)** B-factors calculated from the positional fluctuations of the backbone atoms (red= substrate-free monomer; black= 5′-UMP-bound monomer; green= a substrate-free monomer from the hexamer; blue= a 5′-UMP-bound monomer from the hexamer). **(C)** Dynamical cross-correlation matrices (DCCM) from a 5′-UMP-free monomer, a 5′-UMP-bound monomer, a 5′-UMP-free monomer from the hexamer, and a 5′-UMP-bound monomer from the hexamer.

Table S1. Theoretical masses of putative Nsp15 RNA cleavage products.

	Mass (Da)	m/z (M-H)$^{-}$	m/z (M-2H)$^{2-}$	m/z (M-3H)$^{3-}$
Fl-A-3'OH	804.215	803.207	401.100	267.064
Fl-AA-3'OH	1133.267	1132.259	565.626	376.748
Fl-AAA-3'OH	1462.319	1461.311	730.152	486.432
Fl-AAAU-3'OH	1768.344	1767.336	883.164	588.440
Fl-AAAUA-3'OH	2097.397	2096.389	1047.691	698.125
Fl-AAAUAA-3'OH	2426.449	2425.441	1212.217	807.809
Fl-A-3'P	884.181	883.173	441.083	293.719
Fl-AA-3'P	1213.233	1212.225	605.609	403.403
Fl-AAA-3'P	1542.285	1541.277	770.135	513.087
Fl-AAAU-3'P	1848.310	1847.302	923.147	615.096
Fl-AAAUA-3'P	2177.363	2176.355	1087.674	724.780
Fl-AAAUAA-3'P	2506.415	2505.407	1252.200	834.464
Fl-A-2'3'-cP	866.171	865.163	432.078	287.716
Fl-AA-2'3'-cP	1195.223	1194.215	596.604	397.400
Fl-AAA-2'3'-cP	1524.275	1523.267	761.130	507.084
Fl-AAAU-2'3'-cP	1830.300	1829.292	914.142	609.092
Fl-AAAUA-2'3'-cP	2159.353	2158.345	1078.669	718.777
Fl-AAAUAA-2'3'-cP	2488.405	2487.397	1243.195	828.461
Fl-AAAUAA-TAMRA	3294.734	3293.726	1646.359	1097.237
5'HO-AAAUAA-TAMRA	2757.615	2756.607	1377.800	918.197
5'HO-AAUAA-TAMRA	2428.563	2427.555	1213.274	808.513
5'HO-AUAA-TAMRA	2099.510	2098.502	1048.747	698.829
5'HO-UAA-TAMRA	1770.458	1769.450	884.221	589.145
5'HO-AA-TAMRA	1464.433	1463.425	731.209	487.137
5'HO-A-TAMRA	1135.381	1134.373	566.683	377.453
5'P-AAAUAA-TAMRA	2837.581	2836.573	1417.783	944.853
5'P-AAUAA-TAMRA	2508.529	2507.521	1253.257	835.169
5'P-AUAA-TAMRA	2179.476	2178.468	1088.730	725.484
5'P-UAA-TAMRA	1850.424	1849.416	924.204	615.800
5'P-AA-TAMRA	1544.399	1543.391	771.192	513.792
5'P-A-TAMRA	1215.347	1214.339	606.666	404.108

Table S2. Binding free energies estimated using MMGBSA calculations for the 50-ns segments of the final 450 ns of the 5′-UMP-bound Nsp15 hexamer simulation.

Time (ns)	Nsp15 protomers of the hexameric assembly					
	A	B	C	D	E	F
**385-435**	-10.5 ± 3.7	-23.9 ±1.9	-18.6 ± 1.0	-19.1 ± 1.0	-17.7 ± 0.4	-18.7 ± 1.0
**435-485**	4.7 ± 1.9	-15.4 ± 0.9	-18.3 ± 1.0	-15.3 ± 1.4	-19.1 ± 1.1	-14.7 ± 1.1
**485-535**	0.0	-5.4 ± 3.2	-14.6 ± 1.4	-21.3 ± 1.3	-16.7 ± 1.1	-18.4 ± 2.0
**535-585**	0.0	-5.7 ± 2.8	-1.6 ± 1,2	-15.9 ± 1.5	-14.8 ± 1.2	-6.8 ± 3.2
**585-635**	0.0	-8.7 ±3.0	23.2 ± 1.8	-15.6 ± 1.4	-13.2 ± 1.6	-1.4 ± 2.5
**635-685**	0.0	-7.2 ± 2.0	29.4 ± 1.4	-14.2 ± 1.7	-14.7 ± 1.4	4.4 ± 2.0
**685-735**	0.0	-4.3 ± 3.2	34.6 ± 2.0	-16.9 ± 1.0	-8.4 ± 2.5	1.2 ± 3.1
**735-783**	0.0	-1.2 ± 2.2	32.8 ± 2.6	-17.0 ±1.3	-6.2 ± 1.7	-3.3 ± 2.2
**785-835**	0.0	-0.9 ± 2.7	36.1 ± 2.7	-16.7 ± 0.9	-8.2 ± 2.8	-1.8 ± 1.3

Movie S1. Cryo-EM reconstruction of nucleotide bound Nsp15.

Movie S2. 3D Variability of Apo-H235A-Nsp15 dataset i

Movie S3. 3D Variability of Apo-wt-Nsp15

Movie S4. 3D Variability of Apo-H235A-Nsp15 dataset ii

Supplemental References

1. S. H. Scheres, S. Chen, Prevention of overfitting in cryo-EM structure determination. *Nat Methods* **9**, 853-854 (2012).
2. P. B. Rosenthal, R. Henderson, Optimal determination of particle orientation, absolute hand, and contrast loss in single-particle electron cryomicroscopy. *J Mol Biol* **333**, 721-745 (2003).
3. A. Punjani, J. L. Rubinstein, D. J. Fleet, M. A. Brubaker, cryoSPARC: algorithms for rapid unsupervised cryo-EM structure determination. *Nat Methods* **14**, 290-296 (2017).
4. J. Pei, B. H. Kim, N. V. Grishin, PROMALS3D: a tool for multiple protein sequence and structure alignments. *Nucleic Acids Res* **36**, 2295-2300 (2008).
5. A. M. Waterhouse, J. B. Procter, D. M. Martin, M. Clamp, G. J. Barton, Jalview Version 2--a multiple sequence alignment editor and analysis workbench. *Bioinformatics* **25**, 1189-1191 (2009).
6. S. A. McLuckey, G. J. Van Berkel, G. L. Glish, Tandem mass spectrometry of small, multiply charged oligonucleotides. *J Am Soc Mass Spectrom* **3**, 60-70 (1992).
